# Geometric epitope and paratope prediction

**DOI:** 10.1093/bioinformatics/btae405

**Published:** 2024-07-10

**Authors:** Marco Pegoraro, Clémentine Dominé, Emanuele Rodolà, Petar Veličković, Andreea Deac

**Affiliations:** Department of Computer Science, Sapienza University of Rome, 00185, Italy; Gatsby Computational Neuroscience Unit, University College London, W1T 4JG, United-Kingdom; Department of Computer Science, Sapienza University of Rome, 00185, Italy; Google-DeepMind, 6-8 Handyside Street, London N1C 4UZ, UK; Département d’informatique et de recherche opérationelle, Université de Montréal, QC H2S 3H1, Canada

## Abstract

**Motivation:**

Identifying the binding sites of antibodies is essential for developing vaccines and synthetic antibodies. In this article, we investigate the optimal representation for predicting the binding sites in the two molecules and emphasize the importance of geometric information.

**Results:**

Specifically, we compare different geometric deep learning methods applied to proteins’ inner (I-GEP) and outer (O-GEP) structures. We incorporate 3D coordinates and spectral geometric descriptors as input features to fully leverage the geometric information. Our research suggests that different geometrical representation information is useful for different tasks. Surface-based models are more efficient in predicting the binding of the epitope, while graph models are better in paratope prediction, both achieving significant performance improvements. Moreover, we analyze the impact of structural changes in antibodies and antigens resulting from conformational rearrangements or reconstruction errors. Through this investigation, we showcase the robustness of geometric deep learning methods and spectral geometric descriptors to such perturbations.

**Availability and Implementation:**

The python code for the models, together with the data and the processing pipeline, is open-source and available at https://github.com/Marco-Peg/GEP.

## 1 Introduction

Identifying the binding sites of antibodies is essential for developing vaccines and synthetic antibodies. These binding sites, called paratopes, can bind to antigens, wherein the corresponding binding site is known as the epitope, thus neutralizing harmful foreign molecules in the body. Experimental methods for determining the residues that belong to the paratope and epitope are time-consuming and expensive, highlighting the need for computational tools to facilitate the rapid development of therapeutics. The recent COVID-19 epidemic highlighted this need further, as mutations in the antigen were shown to impact the binding mechanism, potentially reducing the efficacy of existing treatments (Thomson *et al.* 2021). Predicting the binding sites of an antibody-antigen interaction requires considering the entire antigen for epitope prediction and a localized region of the antibody, known as the complementarity-determining region (CDR), for paratope prediction.

**Figure 1.**
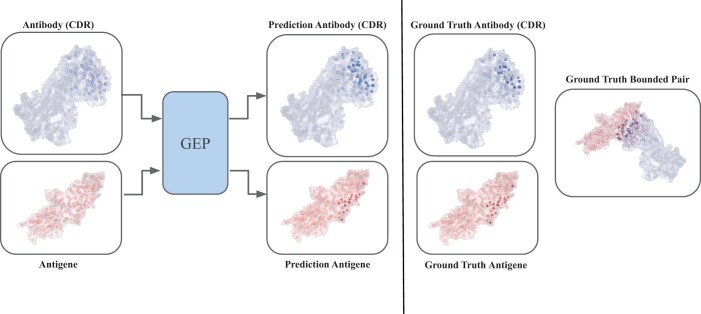
The GEP model processes an unbound antibody–antigen pair as input, predicting the probability of each residue binding with the counterpart molecule. Predicted binding residues are visually emphasized by colored circles (blue for antibody CDR and red for the antigen), with the filled circles indicating the predicted residues. The corresponding bound pair is illustrated on the right.

The shape and structure of molecules play a crucial role in determining their interactions with other molecules, as complementary geometric shapes are required for successful binding (Fischer 1894). The use of geometrical information is further justified by the emergence of technology predicting the single-protein structure, such as Alpha-Fold 2 (Jumper *et al.* 2021), which has comparable accuracy to experimental methods. The integration of geometric and structural information in protein-to-protein interaction studies has led to significant progress (Stärk *et al.* 2022, Dai and Bailey-Kellogg 2021). While several methods have concentrated on the 3D graph representation, few methods (Dai and Bailey-Kellogg 2021, Zhang *et al.* 2023) have investigated the 3D surface representation. We aim to assess the impact of utilizing the geometric representation of the antigen and antibody in the task of epitope-paratope prediction. Our approach, GEP (geometric epitope–paratope) prediction, proposes different geometric representations of the molecules to create accurate predictors for predicting antibody-antigen binding sites (Fig. 1). In particular, we recognize the importance of the outer surface of a molecule in molecular interactions.

Our article introduces several contributions, including the analysis of the importance of geometric information within graph learning using equivariant layers for improved predictions. Moreover, we fully leverage molecular geometric information by representing molecules as surfaces and employing spectral geometry techniques, leading to state-of-the-art performance. Additionally, we will provide a novel dataset and a processing pipeline for PDB molecules, offering molecular representations in both graph and surface formats, facilitating comprehensive cross-method comparisons. The code for the models and the processing pipeline is open-source and available at https://github.com/Marco-Peg/GEP

## 2 Related work

The structure of proteins provides crucial information about the location and orientation of the binding sites. Various approaches have been taken in the literature to address the task of epitope and paratope prediction, including sequential (Liberis *et al.* 2018, Deac *et al.* 2019) and structural (Krawczyk *et al.* 2014, Del Vecchio *et al.* 2022) methods. Furthermore, geometric deep learning has emerged as a powerful tool for predicting protein-protein interactions (Isert *et al.* 2023), with graph-based representations being one of the most common approaches (Tubiana *et al.* 2022, Stärk *et al.* 2022). These methods leverage the geometric information of the molecules to learn complex relationships between epitopes and paratopes. For instance, some approaches (Del Vecchio *et al.* 2022, da Silva *et al.* 2022) use the graph structure to compute features based on neighboring residues, which are then aggregated to highlight the most probable region of interaction.

An alternative approach is to represent proteins as surfaces. MaSIF (Gainza *et al.* 2020) focuses on the more general problem of protein interaction region prediction and uses a surface representation learned through convolutions defined on the surface. PiNet (Dai and Bailey-Kellogg 2021) represents the protein surface as a point cloud and employs PointNet (Qi *et al.* 2017) to classify points as interacting or not. On the contrary, Zhang *et al.* (2023) model the surface of a molecule as a graph and apply an equivariant graph neural network [EGNN, (Satorras *et al.* 2021)] for binding site prediction.

Integrating structural and geometric information has proven to be a promising approach for improving protein interaction prediction. Still, few studies have focused on the specific case of epitope and paratope prediction (Cia *et al.* 2023). Our work supports this view by showing that considering the problem as a geometric one can effectively improve performance.

## 3 Motivation

The interactions between molecules are significantly influenced by the configuration and arrangement of their structures, as successful binding relies on the compatibility of geometric shapes, as noted by Fischer in 1894 (Fischer 1894). To accurately predict molecular interactions, it is essential to incorporate geometric information such as 3D coordinates and spectral descriptors. Our approach to predicting molecular interactions integrates this geometric information into the representation of proteins as graph residues, resulting in a more enhanced and accurate representation. Furthermore, we recognize the importance of the outer surface of a molecule in molecular interactions. To address this, we focus on computations performed on the outer surface of the molecule and then map these predictions to the corresponding residues. By considering the surface of the molecule, we gain valuable insights into the molecular interactions occurring on the surface and enable the use of geometric deep-learning models to analyze these interactions. This approach can potentially provide significant benefits over traditional methods, ultimately leading to more accurate and efficient predictions of molecular interactions.

## 4 Data

We collected a novel dataset of 235 antibody–antigen complexes, with 186 for training and 49 for testing. The training set is taken from the Epipred dataset (Krawczyk *et al.* 2014), while the test set has been selected from the SabDab database (Dunbar *et al.* 2014), ensuring that no more than 70% pairwise sequence identity similarity existed with the train set. We opted for this threshold based on analogous studies (Krawczyk *et al.* 2014, Pittala and Bailey-Kellogg 2020, da Silva *et al.* 2022, Yin and Pierce 2024) in antibody and antigen, where a minimum similarity cutoff of 70% on the antigen was commonly employed. Notably, our similarity assessment encompassed both the antibody’s chains and antigen, thereby imposing a stringent criterion. This criterion on the sequence similarity was chosen to assess the model’s generalization capability effectively.

Additionally, we utilized a separate validation set consisting of 25 antibody–antigen complexes derived from a subset of the Docking Benchmark v5 (Vreven *et al.* 2015), as in Pittala and Bailey-Kellogg (2020). The selected set has at most 91% pairwise sequence identity similarity with the training set.

We further test our models on sequences obtained from the SabDab database and generated by Alpha-Fold (Yin and Pierce 2024). In particular, we constructed two subsets of Protein Data Bank (PDB) complexes based on their accuracy according to Critical Assessment of Predicted Interactions (CAPRI) (Lensink *et al.* 2020) criteria: one subset containing 48 complexes with acceptable accuracy and at most 75% pairwise sequence identity similarity with the training set, and another subset comprising 42 complexes with medium and high accuracy and at most 80% pairwise sequence identity similarity.

### 4.1 Data representation

Comparing methods across different molecular representations is crucial for advancing research in molecular modeling. We developed a reusable pipeline that generates a dataset to evaluate methods using inner and outer structure representations. For each protein, we construct a residue graph (Fig. 4b), representing residues as nodes and establishing edges between the 15 nearest neighboring residues within a 10 Å radius. Each residue is characterized by a 28-dimensional physicochemical feature vector. This vector encompasses a one-hot encoding of the amino acid, encompassing 20 possible types along with one for an unclassified type. Additionally, seven other features are included that portray the physical, chemical, and structural attributes of the amino acid type (see Supplementary data). These supplementary features can be viewed as a consistent embedding, as outlined in Meiler *et al.* (2001).

For each protein, we generated a surface mesh (Fig. 4d) using the PyMOL API with a 1.4 Å water probe radius. We associated each point on the protein’s surface with a residue by finding the closest atom to that point. This association was then used to transfer the feature of each residue to the points on the surface.

## 5 Method

In our experiments, we considered two scenarios: a protein represented through its inner structure (I-GEP) and outer structure (O-GEP). In both cases, we leverage the geometric information to improve the performance of epitope and paratope prediction methods. Details on the methods, including the detailed model architectures, are reported in the Supplementary data.

### 5.1 I-GEP

Our I-GEP model is a method for predicting epitopes and paratopes using a graph-based approach that captures the inner structure of a protein. The I-GEP model has two main components: a structural module that computes an embedding for each residue using the graph structure and a graph attention layer (GAT) that combines information from both the antigen and antibody residues. It’s crucial to note that our model processes these protein pairs without any prior knowledge of their interactions.

**Figure 2.**
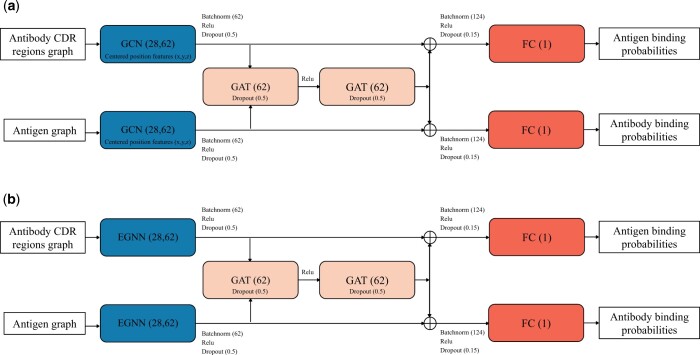
Models architecture: The layers or modules are depicted using color-coded blocks, with the text inside indicating the respective layer type. In parentheses, we provide the dimensions for each layer: GCN, *E*(*n*) invariant layer (EGNN), Graph Attention Layer (GAT), and FC. The arrows indicate the data flow from one module to the next. Additional details about the transformation performed on the input are written. (a) GCN I-GEP. (b) EGNN I-GEP

Specifically, the representation of the antibody and antigen is passed through a structural module summarizing the features of each molecule. We implement this module using a graph convolution layer (GCN), see Fig. 2a. After passing through batch normalization, ReLU activation, and a dropout layer, the distinct features from both the antibody and antigen are fused through a two-layer GAT. Finally, the outputs of the GCN and the two graph attention layers are concatenated to generate two separate predictions, one for the antigen and another for the antibody using a fully connected layer (FC), as shown in Fig. 2.

To improve the accuracy of our predictions, we integrate geometric information into the I-GEP model using two different approaches. In the first approach, EPMP_*xyz*_, we use graph convolutional network layers in the structural module as in EPMP (Del Vecchio *et al.* 2022), but we include the centered 3D coordinates of residues in the input features. The second approach, *E(n)-EPMP*, uses the *E*(*n*) invariant layer encoder from EGNN (Satorras *et al.* 2021) instead of graph convolutional networks. This approach considers only the distances between residues, making it invariant to translations, rotations, and reflections on the residue positions in each molecule. We visually represent this pipeline in Fig. 2a and b.

### 5.2 O-GEP

**Figure 3.**
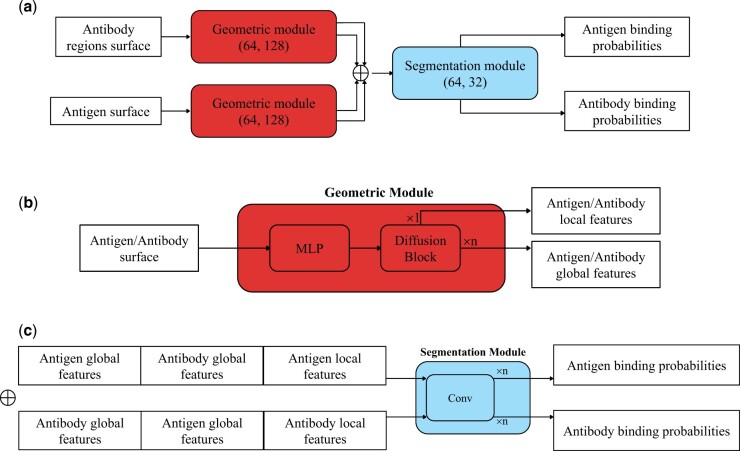
Our model architecture is represented with arrows indicating data flow between modules, using colour-coded blocks to represent layers or modules, with text inside each block specifying the layer type. The model takes antibody-antigen pairs as input, featuring surface point-level features, and produces binding probabilities for each input point. (a) Overall structure of the O-GEP model. (b) Geometric module: The protein representation is first passed through an MLP layer before entering the diffusion block as defined in Sharp et al. (2022). The local and global features are computed by applying the diffusion block a single and *n* times, respectively. (c) Segmentation module: The output of the geometric module is concatenated into two vectors for the antigen and the antibody, respectively. These representations are then sent through the segmentation module to output the binding prediction on the antigen and antibody, respectively. The segmentation module is shared across the two representations and consists of convolutional layers.

Our O-GEP model operates on the protein’s surface and includes a geometric module that uses the surface’s geometry to spread information across it. This process generates features that are then combined and shared between the antibody and antigen through fully connected layers (segmentation module), resulting in an interaction probability for each point on the surface, as shown in Fig. 3a. The O-GEP model is an extension of the architectural foundation established in Pinet, where models store a set of features at each point on the protein surface and generate a binding probability for each point. The input features are computed as explained in and transferred to the surface representation, allowing close comparison with the two geometric representations. We note that the labels of the CDRs are not used as input for the O-GEP methods.

In Pinet, the process begins by individually processing both the antigen and antibody’s surface as a point cloud. This process is iteratively applied to generate two representations: a local representation after one iteration and a global representation after multiple iterations, where local features of each point are pooled into a single vector. Once both proteins have undergone this treatment that we refer as the geometric module in Fig 3b, the local surface features of each protein and their global protein features are combined and subjected to further segmentation through a set of 1D convolution neural network. Importantly, the trainable weights for canonical transformations, local feature extraction, and global feature extraction are shared between the two proteins, as in Dai and Bailey-Kellogg (2021). Finally, the models predict a binding probability for each point on the point cloud.

In modifying this architecture, we explore two different models for the geometric module. As a baseline, we use PointNet (Qi *et al.* 2017) to recreate the architecture proposed in PiNet (Dai and Bailey-Kellogg 2021). In particular, a Spatial Transformation Network ensures invariance to rigid-body transformations for each protein. Subsequently, a multi-layer perceptron (MLP) extracts local surface characteristics. These local surface features are combined into a comprehensive protein feature vector. The second model employs diffusion layers from DiffNet (Sharp *et al.* 2022) to propagate features on the surface. This change is advantageous because DiffNet can compute features on both point clouds and meshes independently. This makes our model robust against surface perturbations and suitable for handling meshes and point clouds with fewer points.

We further examine the impact of using the Heat Kernel Signature (HKS) as an extra geometric descriptor input. The HKS (Sun *et al.* 2009) is a concise point-wise spectral signature which summarizes local and global information about the intrinsic geometry of a shape by capturing the properties of the heat diffusion process on the surface. One of the key benefits of using HKS is that it remains stable even under minor surface perturbations, thus enabling it to withstand even conformational rearrangements of the proteins. To utilize the HKS descriptor, we concatenate it with the input features at each point on the surface and then pass the concatenated data through the geometric module.

To transfer the binding probabilities from the protein’s surface to the residues, we utilized the average of all the points on the surface that correspond to the same residues. This method ensures that the binding probabilities are accurately represented in the residue space, enabling us to make reliable predictions about epitope and paratope locations.

### 5.3 Training and evaluation

The networks were trained using the class-weighted binary cross-entropy loss and the Adam stochastic gradient descent (SGD) optimizer to handle imbalanced binary classification tasks. To enhance model robustness, we applied random rotations to dataset instances. We report training details in the Supplementary data.

Given the significant disparity in class sizes, we utilize Matthew’s correlation coefficient (MCC) between the residues’ classification as our main benchmarking metric for model evaluation. This aligns with evaluation methods in similar studies such as (Krawczyk *et al.* 2014, Cia *et al.* 2023). We also report the area under the receiver operating characteristic curve (AUC ROC) and the area under the precision–recall curve (AUC PR) as used in (Dai and Bailey-Kellogg 2021, Del Vecchio *et al.* 2022). All reported values are aggregated across five random seeds to ensure the robustness of our findings.

## 6 Results

In this section, we report the results of our experiments and demonstrate the contribution of geometric information on the task of epitope-paratope prediction.

### 6.1 I-GEP results

We conducted experiments to evaluate the effectiveness of incorporating geometric information by comparing our proposed models from section with the EPMP model proposed in Del Vecchio *et al.* (2022). The results obtained from the test set, which includes complexes with a pairwise sequence identity similarity of at most 70%, are presented in Supplementary Table 1a. The metrics demonstrate that adding geometric information leads to increased performance. Specifically, the use of the *E*(*n*) invariant layer (*E*(*n*)-EPMP) resulted in an improvement in the AUR ROC and AUR PR metrics for both antibody and antigen.

### 6.2 O-GEP results

To test the performance of O-GEP models, we consider the methods proposed in Section with different combinations of input features. In addition to the physicochemical features, we test different combinations of geometric information: 3d coordinates (xyz) and HKS. For the DiffNet models, we consider both the point cloud (_*pc*_) and the mesh (_*m*_) of the surface.

The results obtained from the test set are summarized in Supplementary Table 1b. Notably, the HKS emerges as a valuable feature for both paratope and epitope predictions. Its incorporation into the PiNet baseline leads to an improvement in performance for both tasks, with an increase of at least 0.03 in the MCC metric. Of particular interest are the results obtained from the DiffNet_*pc*_ (hks) and DiffNet_*m*_ (hks) models in antibody predictions. Here, the addition of HKS significantly enhances the MCC score by 0.09 and 0.12, respectively, compared to the DiffNet models without this feature. Conversely, the absence of coordinates as input appears to diminish the performance of antigen predictions. The effectiveness of surface features like the HKS in paratope prediction can be attributed to the localized and specific structural characteristics captured, especially within the protein’s Complementarity Determining Regions (CDRs).

Interestingly, aside from the sole input of HKS, the PiNet and DiffNet models perform similarly. The DiffNet model with only 3D coordinates as input [DiffNet (xyz)] outperforms its PiNet counterpart [PiNet (xyz)] and performs at comparable levels to PiNet with the additional HKS feature [PiNet (xyz+hks)]. However, the combined use of both 3D coordinates and HKS does not yield the same performance improvement in epitope prediction for DiffNet as observed in PiNet. This phenomenon may suggest that the heat diffusion process modeled by DiffNet sufficiently approximate the information provided by HKS on the antigen, rendering the additional HKS features redundant in the model.

Regarding the choice between mesh representation and point cloud, it is essential to note that when input features are consistent, the performance difference between the two representations is relatively small, typically within a range of 0.4 and usually overlapping in our experiments (see Supplementary Table 1b). Factors contributing to this slight difference may include the computation of eigenvectors and the inherent structure of the representations themselves. Point cloud representation offers greater flexibility as it does not impose connectivity constraints on neighboring nodes, allowing for a more adaptable representation for proteins.

### 6.3 Qualitative results

**Figure 4.**
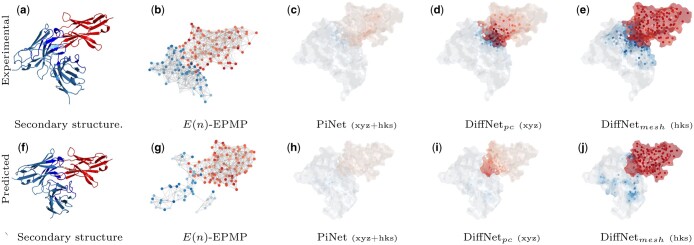
Qualitative comparison between experimental and Alpha-Fold 2 predicted complex ‘7e9b’. The continuous binding predictions are represented as a color gradient in blue and red for the antigen and antibody, respectively. (a) Secondary structure, (b) *E*(*n*)-EPMP, (c) PiNet (xyz+hks), (d) DiffNet_*pc*_ (xyz), (e) DiffNet_*mesh*_ (hks), (f) secondary structure, (g) *E*(*n*)-EPMP, (h) PiNet (xyz+hks), (i) DiffNet_*pc*_ (xyz), and (j) DiffNet_*mesh*_ (hks)

We also conducted a qualitative assessment of our methods. In Fig. 4, we show the predicted binding on the complex “7e9b” for the best model in Supplementary Table 1b. In the Supplementary data, we provide more qualitative examples of all the methods considered.

We observed distinct behavior in epitope and paratope prediction when visualizing binding probability on graph nodes using color gradients in I-GEP models (Fig. 4g and b). Paratope prediction focuses on residues closest to the antigen, while epitope prediction primarily targets sparse regions of the antigen, often its spiky edges. This is translated in the I-GEP results in better predictive performance for the antibody.

In O-GEP models, predictions are visualized on the protein surface and residues. Predictions are highly localized on the region nearest to the binding molecule, especially for the epitope(see Fig. 4c and d). However, HKS alone may not be sufficient to propagate information globally across the antigen, leading to high binding predictions across all geometry of the antigen (see Fig. 4e and j).

### 6.4 I-GEP and O-GEP comparison

Our study indicates that diverse geometric representations hold utility across various tasks. Surface-based models excel in predicting antigen binding, whereas graph models demonstrate superior performance in antibody prediction. This divergence can be attributed to the local nature of predicting the antibody binding region, primarily concentrating on the CDR, a well-structured and localized area. In contrast, epitope prediction requires a more global perspective, hence favoring a surface-based approach.

As I-GEP and O-GEP yield complementary results across various tasks, we decided to combine their predictions. In the Supplementary data, we present our approach, which involves combining the predictions made by I-GEP and O-GEP using simple methods such as taking the mean or product of the predictions for each residue. Remarkably, the results reveal that the performance of these mixed models can either match or even surpass that of the individual models. This finding underscores the potential effectiveness of combining different processing methods.

## 7 Structural variations

Proteins, including antibodies and antigens, are inherently flexible and dynamic entities. Their shapes can undergo changes in response to alterations in their environment or other factors, resulting in various conformations for the same amino acid sequence. In this section, we analyze how these variations in shape influence the prediction of epitopes and paratopes in GEP.

### 7.1 Unbounded complexes

The structure and geometry of the proteins might change depending on whether they are bound or not. We compare the results between bound and unbound structures for the antibody and antigen complexes on the validation set derived from a subset of the Docking Benchmark v5 (Vreven *et al.* 2015). This benchmark provides protein complexes both in unbound and bounded conditions, allowing us to compare the two settings. We computed the mean root-mean-square deviation (mRMSD) between corresponding complexes in the two datasets and find out that the antigens have a mRMSD 3.67 Å, while the antibody a mRMSD of 3 Å.

Supplementary Table 2a and c shows the results of the I-GEP and O-GEP models, respectively, on the validation set used during training. Since the validation set shares a much higher identity similarity with the training set with respect to that test set used in Supplementary Table 1, the performances are much higher. The O-GEP models are able to reach an MCC of 0.54 and an AUC ROC of 0.91 on the antigene, while the *E*(*n*)-EPMP reaches an MCC of 0.46 and an AUC ROC of 0.83 on the antibody. As in Supplementary Table 1, the addition of the HKS features increases the performance of the PiNet model, while the *E*(*n*)-EPMP stays as the best I-GEP model. When we compare the bounded conformations (Supplementary Table 2a and c) with the unbounded counterpart (Supplementary Table 2b and d), we can notice a constant reduction in the performance, but the relative performance trends remain consistent. In the Supplementary data, we plot qualitative examples of the ’2fd6’ both in the bounded and unbounded setting.

#### 7.1.1 Alpha-Fold predictions

In the case where the experimental structure is not available, it is possible to use tools such as Alpha-Fold 2 (Jumper *et al.* 2021) to predict the structure from the protein sequences.

We test our model on Alpha-Fold predictions from Yin and Pierce (2024) where the geometry of the proteins might also change slightly due to reconstruction error. In Supplementary Table 4, we selected the reconstructions that were measured to have high and medium CAPRI accuracy. The complexes have a RMSD of 11.25 Å for the antigens and of 1.35 Å for the antibody. In the Supplementary data, we also report and discuss the results of acceptable accuracy reconstruction.

The Alpha-Fold results in Supplementary Table 3 consistently exhibit lower values compared to those from the experimental complexes. This can be attributed to the visibly distinct structure of the reconstructed antibody in Fig. 4g. Nevertheless, looking at the qualitative results in Fig. 4, results remain notably promising, with predictions highly localized on the region nearest to the binding molecule for both paratope and epitope. In the Supplementary data, we provide the qualitative examples of all the GEP methods.

#### 7.1.2 Discussion

Both the unbounded and Alpha-Fold datasets introduce changes or errors in geometry. Generally, the models perform less effectively on these datasets. However, we assess the robustness of geometric models relative to the original by examining the Wasserstein distances (WD) between the performance with the original data and the geometric altered data set. We present a table of results for all models in the Supplementary material. In the O-GEP models, we observe in both sets, that the HKS is as a valuable feature for epitope, decreasing the WD leading to more robust model. This observation aligns with the enhanced performance of models incorporating these features shown in Supplementary Table 1. In the I-GEP models, the inclusion of the equivariant layer results in a lower WD for the antibody, signifying stronger robustness to geometric changes compared to the original models. This aligns with the observation that I-GEP models excel in predicting the paratope due to their local structure. Comparing the two sets, the Alpha-Fold complexes exhibit a higher RMSD for the antigens than the unbound set. This suggests that the Alfa-Fold structures deviate more geometrically from the original protein than the unbound structures. The results corroborate this, as the WD on the Alpha-Fold set is larger than that on the unbounded structures.

## 8 Conclusions

We investigated the effectiveness of geometric deep learning techniques in predicting antibody-antigen interactions. Our results indicate that incorporating geometric information is crucial for accurately predicting epitope and paratope regions. Specifically, the use of an invariant representation in I-GEP models improve previous models, and O-GEP models with diffusion layers and additional geometric features achieved state-of-the-art performance. Inspired by the complementary roles of these two approaches, we explored the combination of their predictions, yielding enhanced performance. This combination of methodologies presents an intriguing avenue for further investigation in epitope and paratope predictions, showcasing the potential of leveraging diverse computational techniques to augment predictive capabilities. Moreover, our study has shed light on the impact of geometric variations arising from conformational changes or reconstruction errors. Despite these challenges, our models have shown better results compared to previous methods, underscoring the robustness of our approach. Notably, our observation that the HKS provides geometric information resilient to minor perturbations in protein structures offers promising avenues for future exploration. For this reason, we believe future research could explore using spectral shape analysis to address the more complex problem of conformational rearrangement in antigen-antibody binding. A limitation of our model is that it cannot solely utilize an antigen or antibody as input, as it relies on both proteins’ properties to predict the most suitable interacting region. In our future research works, we aim to explore the significance of geometric information in predicting partner-unspecific interfaces.

## Supplementary Material

btae405_Supplementary_Data
