# Supplementary material for: Geometric epitope and paratope prediction
Source: Bioinformatics. 2024 Jul 10;40(7):btae405. doi: 10.1093/bioinformatics/btae405 (PMC11245313; doi:10.1093/bioinformatics/btae405)
Supplement: btae405_Supplementary_Data [file btae405_supplementary_data.zip › supp/suppmat_compressed.pdf]

Journal Title Here, 2022, pp. 1–5

doi: DOI HERE

Advance Access Publication Date: Day Month Year

Paper

PAPER

Geometric Epitope and Paratope Prediction Supplementary material

Marco Pegoraro,[1,*] Clémentine Dominé,[2] Emanuele Rodolà,[1] Petar Veličković[3] and Andreea Deac[4]

[1] Sapienza, University of Rome, 5 Piazzale Aldo Moro, 00185, , Italy, [2]Gatsby Computational Neuroscience Unit, University College London, 25 Howland St, W1T 4JG, United-Kingdom, [3]Google-DeepMind, Handyside Street, N1C 4UZ, United-Kingdom and [4]Mila, Université de Montréal, 6666 Rue Saint-Urbain, QC H2S 3H1, Canada

[*]Corresponding author. pegoraro@di.uniroma1.it

FOR PUBLISHER ONLY Received on Date Month Year; revised on Date Month Year; accepted on Date Month Year

Abstract

In our work's Supplementary material, we provide additional information on the model's architecture and feature selection. We also provide additional images of our method's prediction.

Key words: Paratope-Epitope prediction, Geometric Deep Learning, Drug Discovery

Data

Data Availability

Training models for predicting protein interactions, especially between antigens and antibodies, is constrained by the scarcity of protein complexes. Antigen/antibody complexes are a notably small fraction of the total protein complexes known. The experimental challenge in acquiring structural data for these complexes leads to a limited dataset available for training. This shortage of data introduces several challenges for machine learning methodologies, including the risk of overfitting, which compromises the models' ability to generalize. This issue is particularly acute in the context of epitope prediction. Given that any area on an antigen could potentially serve as an epitope and that antigens can have multiple epitopes, the solution space is vast and high-dimensional.

Data Selection

We collected a novel dataset for our experimentation.

Train set: The train set is made of 186 [Dunbar et al., 2014] protein complexes that result from the concatenation of the training, validation and test set of the PECAN datasets. The Chothia numbered PDB files were obtained from the Sabdab database.

Test set: The test set is made of 49 protein complexes extracted from the SabDab database and shares no more than 70 % global alignment similarity identity with the train set. The Alpha-Fold2 data set shares the same protein complexes, which allows for comparison. Specifically, 48 PDB structures from AlphaFold2 have acceptable CAPRI ratings on AlphaFold reconstruction and 42 PDB structures from AlphaFold with High-Medium CAPRI ratings on AlphaFold reconstruction. They, respectively, have a maximum global alignment similarity of 75 % and 80%. The CDR loops were defined as residues 24–42 (CDR1), 57–76 (CDR2), and 107–138 (CDR3)),as in previous work Lee et al. [2022].

Validation set: The validation set is made of 25 protein complexes extracted from the Docking Benchmark v5 database and shares no more than 90.33 % global alignment similarity with the train set. The unbounded set shares the same protein complexes which allows comparison. The amino acids identified as part of the heavy chain in the PDB will constitute the CDR, whereas those identified with the antigen species will depict the antigen. We include a table for each set listing the PDB name, along with the corresponding antigen name and species. The CDR region is defined according to the Chotia numbering provided by the SabDab database.

Attached to the Supplementary materials, we provide the csv files with the selected pdb for each set.

Methods

In this Section, we provide a detailed explanation of the models' architecture presented in the paper.

© The Author 2022. Published by Oxford University Press. All rights reserved. For permissions, please e-mail: journals.permissions@oup.com

Layer dimensions

For the EPMP$_{xyz}$ model, we use a graph convolution layer with inner dimension 31 and two GAT layers with inner dimension 62. In contrast, for the $E(n)$-EPMP, we use one $E(n)$-invariant layer with an inner dimension of 28 and two GAT layers with inner dimension 56.

For all the PiNet models, the geometric module comprises two layers with dimensions 64 and 128, while the segmentation module is composed of two layers with dimensions 64 and 32. While for the DiffNet models we used a geometric module with dimensions 32 and 64.

Hyper-parameters

During training, we combined the losses from both tasks, paratope and epitope prediction. Hyperparameter tuning involved a search for the optimal learning rate from the set $\{10^{-2}, 10^{-3}, 5 \times 10^{-3}, 10^{-5}\}$ and kept the model with the best performance on the validation set. After the hyperparameter search, we found that the best learning rates were: 10^{-3} for EPMP and PiNet, 10^{-2} for $E(n)$-EPMP, 5×10^{-3} for DiffNet. We trained the I-GEP model taking as batch 8 antibody-antigen complex at the time, while the O-GEP model was trained taking 16 complexes per batch. The training was performed on a NVIDIA A100-SXM4-40GB. All models were trained for 200 epochs to ensure validation loss saturation, and the weights yielding the best validation metrics during training were selected. We conducted training with five random seeds for each model, evaluating performance using the weights yielding the best validation set results in each run.

The surface generated by PyMOL is composed of around 14k points. To ease and fast the training procedure we subsampled the surface considering only 2k points. In the case of point clouds, we used a random subsampling during training, while for the mesh, we used a simplification method based on surface subdivision loop.

Pairwise Similarity measure

To compute the pairwise similarity between two antibody-antigen complexes, we perform a global alignment of the amino acid sequences of the corresponding chains (Heavy, Light, and Antigen). We count the number of perfectly aligned amino acids between these sequences. Subsequently, this count is divided by the length of the shortest sequence, resulting in a similarity score ranging from 0 to 1.

Wasserstein distance

To evaluate the robustness of the proposed models, we compute the Wasserstein distance between the sets of results obtained from a batch of runs. Given two sets of results $\{U_1, ..., U_n\}$ and $\{V_1, ..., V_n\}$, the 1D Wasserstein distance [Ramdas et al., 2017] can be defined as:

$$W_1(\{U_1, ..., U_n\}, \{V_1, ..., V_n\}) = \int_{-\infty}^{+\infty} |\mathbf{U} - \mathbf{V}| \tag{1}$$

where $\mathbf{U}$ and $\mathbf{V}$ are the Cumulative Distribution Functions (CDFs) approximated by $\{U_1, ..., U_n\}$ and $\{V_1, ..., V_n\}$, respectively. In our code we use the implementation provided by the Python library scipy.

Features

A 28-dimensional physicochemical feature vector characterizes each residue. This vector encompasses a one-hot amino acid encoding, encompassing 20 possible types and one for an unclassified type. The last seven features represent summaries for each amino acid:

- **Steric Parameter:** This feature characterizes the size and spatial arrangement of the amino acid. It relates to the bulkiness or the amount of space an amino acid occupies within a protein structure.
- **Polarisability:** Polarisability reflects an amino acid's ability to undergo induced electric polarization when exposed to an electric field. It provides information about the flexibility and response of the amino acid to its environment.
- **Volume:** This feature quantifies the molecular volume of an amino acid. It represents the space an amino acid occupies in a three-dimensional context and can be crucial in understanding the packing of amino acids within a protein structure.
- **Hydrophobicity:** Hydrophobicity indicates how hydrophilic or hydrophobic an amino acid is. It plays a fundamental role in the folding and stability of proteins.
- **Isoelectric Point:** The isoelectric point of an amino acid is the pH at which it carries no net electrical charge. It is a crucial property when considering protein electrophoresis and interactions in various biological processes.
- **Helix Probability:** This feature represents the probability of an amino acid being part of an alpha-helix secondary structure. It gives insights into the structural tendencies of the amino acid within proteins
- **Sheet Probability:** Similarly to the alpha-helix probability, this feature denotes the probability of an amino acid being part of a beta-sheet secondary structure. It provides information about the amino acid's role in forming beta-sheets in protein structures.

These features are vital for understanding and characterizing the properties of amino acids within proteins and can be used in various bioinformatics and structural biology applications [Vreven et al., 2015].

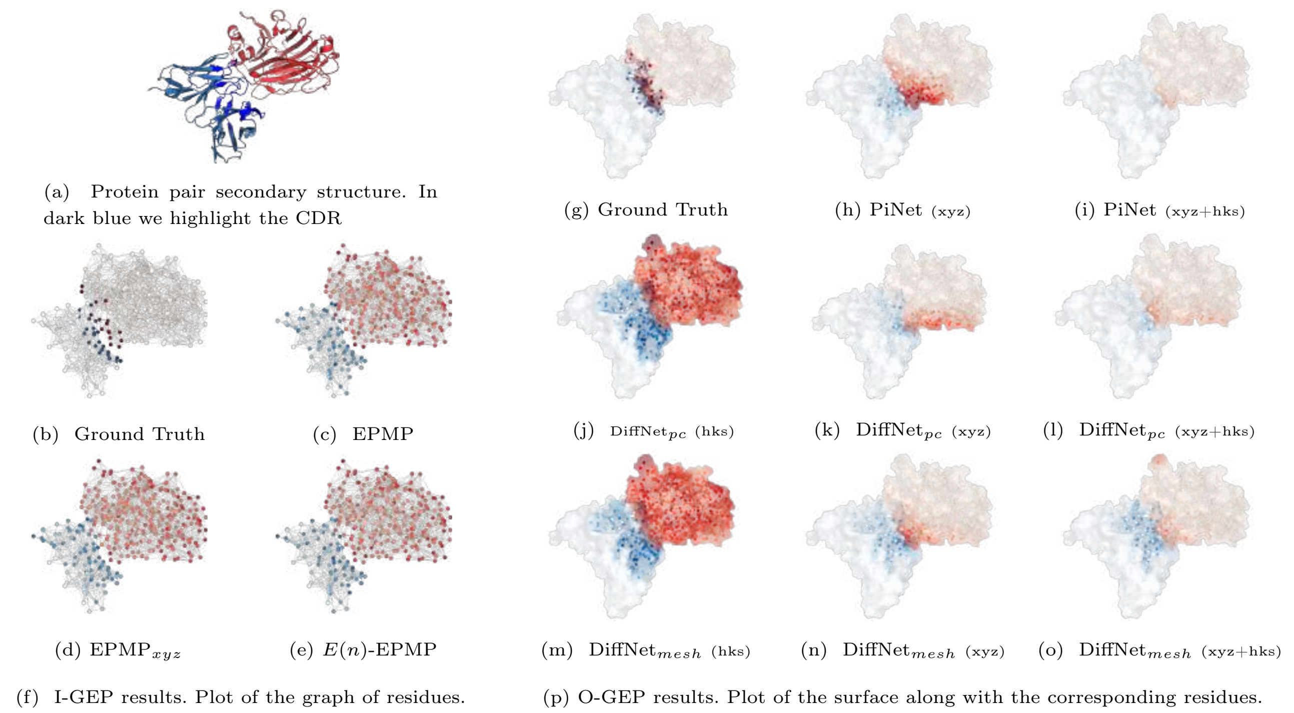

Fig. 1. All O-GEP and I-GEP models results for the antibody-antigen complex '6nyq'. The continuous binding predictions are represented as a color gradient in red and blue for the antigen and antibody, respectively.

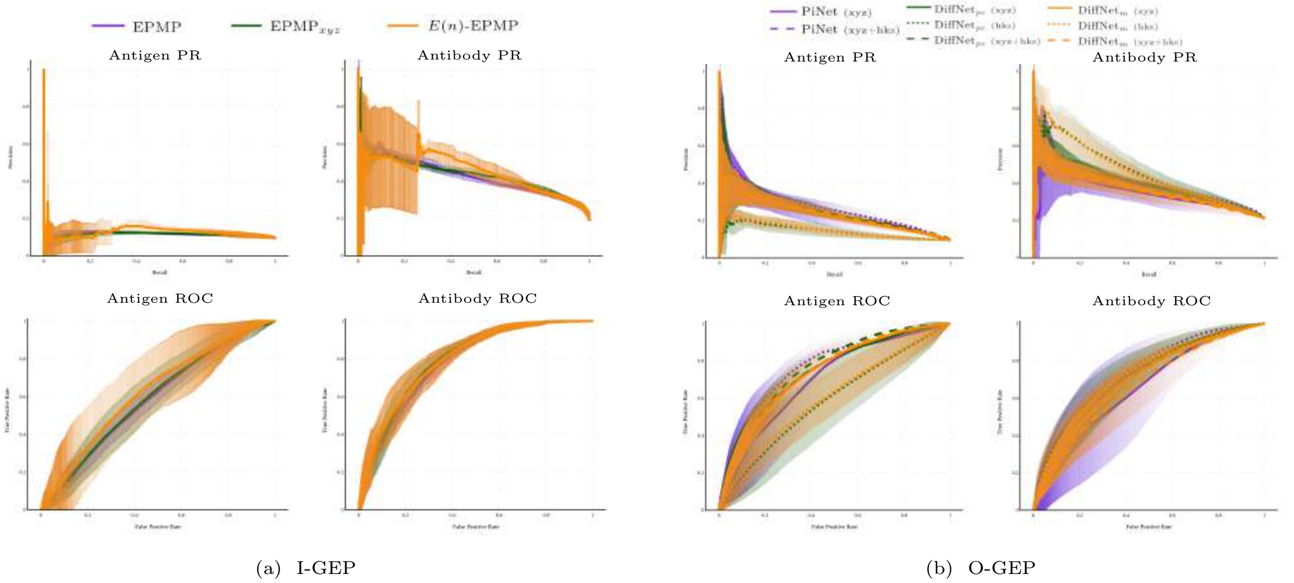

Fig. 2. Receiver operating characteristic (ROC) and Precision-Recall (PR) curves for epitope and paratope prediction. We report the average values with the standard deviation over five runs for all the method presented in Table 1 of the main paper.

Additional results

ROC and PR Curves

We report in figure 2b the Receiver operating characteristic (ROC) and Precision-Recall (PR) curves for epitope and paratope prediction on the test set.

The ROC curve depicted in Figure 2a shows that $E(n)$-EPMP consistently outperforms the other methods. On the O-GEP models, PiNet (xyz+hks) and DiffNet$_{pc}$ (xyz+hks) have the highest ROC curves on the antigen.

Table 1. Quantitative results evaluated on the test set with at most 70% similarity with the addition of the combination between IGEP and OGEP methods referred to as MIXmean and MIXprod. We report the mean and standard deviation (±) over five runs of the Matthew's correlation coefficient (MCC), the area under the receiver operating characteristic curve (AUR ROC), and the area under the precision-recall curve (AUC PR). We write in bold the best results

	Antigen			Antibody		
	MCC	AUC ROC	AUC PR	MCC	AUC ROC	AUC PR
EPMP	0.06 ± 0.01	0.59 ± 0.00	0.11 ± 0.00	0.33 ± 0.01	0.78 ± 0.01	0.42 ± 0.01
EPMP$_{xyz}$	0.03 ± 0.04	0.60 ± 0.01	0.11 ± 0.01	0.34 ± 0.02	**0.80 ± 0.01**	0.43 ± 0.02
$E(n)$-EPMP	0.06 ± 0.04	0.65 ± 0.01	0.14 ± 0.01	0.33 ± 0.02	**0.80 ± 0.02**	0.48 ± 0.02
PiNet (xyz)	0.15 ± 0.08	0.75 ± 0.02	0.26 ± 0.03	0.17 ± 0.11	0.70 ± 0.04	0.40 ± 0.05
PiNet (xyz+hks)	**0.18 ± 0.02**	0.76 ± 0.02	**0.29 ± 0.03**	0.20 ± 0.05	0.74 ± 0.01	0.42 ± 0.03
DiffNet$_{pc}$ (xyz)	**0.18 ± 0.01**	0.76 ± 0.01	0.27 ± 0.01	0.19 ± 0.05	0.72 ± 0.02	0.42 ± 0.03
DiffNet$_{pc}$ (hks)	0.06 ± 0.04	0.59 ± 0.02	0.15 ± 0.02	0.28 ± 0.05	0.75 ± 0.01	0.47 ± 0.02
DiffNet$_{pc}$ (xyz+hks)	0.16 ± 0.04	0.74 ± 0.02	0.24 ± 0.02	0.20 ± 0.02	0.73 ± 0.02	0.44 ± 0.02
DiffNet$_{m}$ (xyz)	0.17 ± 0.03	0.75 ± 0.01	0.26 ± 0.02	0.16 ± 0.04	0.69 ± 0.02	0.40 ± 0.02
DiffNet$_{m}$ (hks)	0.08 ± 0.04	0.61 ± 0.02	0.17 ± 0.02	0.28 ± 0.01	0.74 ± 0.01	0.47 ± 0.01
DiffNet$_{m}$ (xyz+hks)	0.16 ± 0.02	0.74 ± 0.02	0.24 ± 0.02	0.19 ± 0.05	0.71 ± 0.02	0.41 ± 0.03
MIXmean-egnn DiffNet$_{pc}$ (hks)	0.11 ± 0.04	0.63 ± 0.03	0.17 ± 0.02	**0.35 ± 0.04**	**0.80 ± 0.01**	**0.54 ± 0.01**
MIXmean-egnn PiNet(xyz+hks)	**0.18 ± 0.03**	0.72 ± 0.02	0.27 ± 0.02	0.28 ± 0.07	**0.80 ± 0.01**	0.50 ± 0.03
MIXmean-egnn DiffNe$_{pc}$ (xyz)	0.18 ± 0.02	0.74 ± 0.04	0.27 ± 0.01	0.28 ± 0.05	0.79 ± 0.02	0.51 ± 0.02
MIXprod-egnn DiffNet$_{pc}$ (hks)	0.03 ± 0.01	0.63 ± 0.03	0.18 ± 0.01	**0.35 ± 0.03**	**0.80 ± 0.01**	**0.54 ± 0.01**
MIXprod-egnn PiNet (xyz+hks)	0.06 ± 0.04	**0.77 ± 0.03**	**0.29 ± 0.03**	0.14 ± 0.07	0.77 ± 0.02	0.47 ± 0.03
MIXprod-egnn DiffNet$_{pc}$ (xyz)	0.03 ± 0.01	**0.77 ± 0.01**	0.28 ± 0.01	0.15 ± 0.06	0.76 ± 0.01	0.47 ± 0.02

Table 2. Wasserstein distances between the bounded and unbound results. We highlight the lower value for each column in bold.

	Antigen			Antibody		
	MCC	AUC ROC	AUC PR	MCC	AUC ROC	AUC PR
EPMP	0.06	0.04	**0.03**	0.07	0.04	0.10
EPMP$_{xyz}$	0.12	0.07	0.04	0.07	0.04	0.11
$E(n)$-EPMP	0.08	0.02	**0.03**	0.06	0.03	0.09
PiNet (xyz)	0.11	0.07	0.15	0.08	0.03	0.13
PiNet (xyz+hks)	0.08	0.06	0.11	0.07	0.02	0.09
DiffNet$_{pc}$	0.14	0.06	0.18	0.06	**0.01**	0.09
DiffNet$_{pc}$ (hks)	**0.02**	**0.01**	**0.03**	0.09	0.04	0.14
DiffNet$_{pc}$ (xyz+hks)	0.10	0.06	0.12	**0.02**	**0.01**	**0.07**
DiffNet$_{m}$ (xyz)	0.11	0.06	0.13	0.05	0.02	0.10
DiffNet$_{m}$ (hks)	**0.02**	0.02	**0.03**	0.07	0.04	0.14
DiffNet$_{m}$ (xyz+hks)	0.09	0.06	0.11	0.07	0.02	0.10

Combined models

Our research suggests that different geometrical representation information is useful for different tasks. Therefore, we combine the prediction of our method to understand if their strengths can be combined. We use two simple methods for the combination of the prediction, the product and the mean. In table 1, we report the Mixed model prediction's alongside the original results found in the paper for easy visual comparison. The results show that the performance of the mixed models matches or improves on the results of the models alone. Various mixture methods and model combinations result in different levels of performance. Among these, the pairing of $E(n)$-EPMP and DiffNet with HKS and position features proves to be the most efficient blend for enhancing antibody predictions. The pairing of $E(n)$-EPMP and PiNet with HKS features emerges as the best combination for the antigen.

Bounded-Unbounded

We report the qualitative results of unbound and bound structures for the antibody-antigen complexes in Figure 4 and 3. In Table 2 we report the Wasserstein distance between the results on the bounded and unbounded validation set.

Table 3. Quantitative comparison on the predictions performed on a set of SabDab complexes and AlphaFold2-Reconstructed Structures with acceptable accuracy. We report Matthew's correlation coefficient (MCC), area under the receiver operating characteristic curve (AUR ROC), the area under the precision-recall curve (AUC PR). We write in bold the best results

(a) I-GEP on experimental complexes

	Antigen			Antibody		
	MCC	AUC ROC	AUC PR	MCC	AUC ROC	AUC PR
EPMP	0.06 ± 0.01	0.61 ± 0.00	0.12 ± 0.00	**0.36 ± 0.01**	0.80 ± 0.01	0.47 ± 0.01
$EPMP_{xyz}$	0.06 ± 0.03	0.63 ± 0.02	0.13 ± 0.01	**0.36 ± 0.01**	0.81 ± 0.01	0.48 ± 0.01
$E(n)$-EPMP	**0.08 ± 0.05**	**0.69 ± 0.01**	**0.15 ± 0.00**	**0.36 ± 0.02**	**0.82 ± 0.02**	**0.52 ± 0.04**

(b) I-GEP on predicted complexes

	Antigen			Antibody		
	MCC	AUC ROC	AUC PR	MCC	AUC ROC	AUC PR
EPMP	0.04 ± 0.01	0.59 ± 0.00	0.12 ± 0.00	0.02 ± 0.02	0.51 ± 0.01	0.07 ± 0.00
$EPMP_{xyz}$	0.03 ± 0.04	0.60 ± 0.03	0.12 ± 0.01	0.04 ± 0.03	0.53 ± 0.02	0.08 ± 0.01
$E(n)$-EPMP	**0.06 ± 0.04**	**0.64 ± 0.01**	**0.14 ± 0.01**	**0.07 ± 0.04**	**0.61 ± 0.07**	**0.11 ± 0.04**

(c) O-GEP on experimental complexes

	Antigen			Antibody		
	MCC	AUC ROC	AUC PR	MCC	AUC ROC	AUC PR
PiNet (xyz)	0.17 ± 0.10	**0.82 ± 0.02**	0.29 ± 0.03	0.21 ± 0.07	0.71 ± 0.03	0.42 ± 0.04
PiNet (xyz+hks)	0.19 ± 0.03	**0.82 ± 0.01**	0.30 ± 0.01	0.20 ± 0.03	0.73 ± 0.01	0.44 ± 0.02
$DiffNet_{pc}$ (xyz)	0.23 ± 0.06	0.81 ± 0.03	0.29 ± 0.04	0.21 ± 0.05	0.74 ± 0.03	0.45 ± 0.04
$DiffNet_{pc}$ (hks)	0.08 ± 0.02	0.61 ± 0.01	0.13 ± 0.00	**0.33 ± 0.05**	**0.78 ± 0.01**	**0.53 ± 0.02**
$DiffNet_{pc}$ (xyz+hks)	0.24 ± 0.02	0.80 ± 0.01	0.30 ± 0.02	0.21 ± 0.05	0.73 ± 0.02	0.44 ± 0.03
$DiffNet_{m}$ (xyz)	0.23 ± 0.04	**0.82 ± 0.02**	**0.31 ± 0.03**	0.15 ± 0.05	0.69 ± 0.02	0.38 ± 0.02
$DiffNet_{m}$ (hks)	0.07 ± 0.02	0.60 ± 0.01	0.13 ± 0.00	**0.33 ± 0.03**	0.77 ± 0.00	0.52 ± 0.00
$DiffNet_{m}$ (xyz+hks)	**0.25 ± 0.04**	0.81 ± 0.02	**0.31 ± 0.03**	0.22 ± 0.03	0.72 ± 0.02	0.43 ± 0.02

(d) O-GEP on predicted complexes

	Antigen			Antibody		
	MCC	AUC ROC	AUC PR	MCC	AUC ROC	AUC PR
PiNet (xyz)	0.03 ± 0.04	0.58 ± 0.05	0.13 ± 0.02	**0.07 ± 0.07**	**0.66 ± 0.03**	**0.13 ± 0.02**
PiNet (xyz+hks)	0.03 ± 0.03	0.55 ± 0.04	0.12 ± 0.02	0.01 ± 0.04	0.60 ± 0.05	0.09 ± 0.02
$DiffNet_{pc}$ (xyz)	0.05 ± 0.01	0.60 ± 0.04	0.14 ± 0.01	−0.00 ± 0.02	0.57 ± 0.04	0.08 ± 0.01
$DiffNet_{pc}$ (hks)	**0.08 ± 0.02**	0.60 ± 0.01	0.14 ± 0.01	0.05 ± 0.04	0.54 ± 0.04	0.10 ± 0.02
$DiffNet_{pc}$ (xyz+hks)	0.05 ± 0.02	0.61 ± 0.03	0.14 ± 0.02	0.01 ± 0.04	0.63 ± 0.04	0.10 ± 0.01
$DiffNet_{m}$ (xyz)	0.05 ± 0.02	0.61 ± 0.04	0.14 ± 0.02	0.02 ± 0.02	0.61 ± 0.03	0.10 ± 0.01
$DiffNet_{m}$ (hks)	0.06 ± 0.01	0.60 ± 0.02	0.14 ± 0.01	0.04 ± 0.02	0.55 ± 0.02	0.11 ± 0.01
$DiffNet_{m}$ (xyz+hks)	**0.08 ± 0.02**	**0.62 ± 0.04**	**0.16 ± 0.02**	0.05 ± 0.03	0.62 ± 0.01	0.10 ± 0.01

Table 4. Wasserstein distances between the experimental and Alpha-Fold predicted complexes. We highlight the lower value for each column in bold.

(a) High-Medium reconstructions

	Antigen			Antibody		
	MCC	AUC ROC	AUC PR	MCC	AUC ROC	AUC PR
EPMP	**0.01**	**0.02**	0.02	0.32	0.26	0.36
$EPMP_{xyz}$	**0.01**	0.03	0.01	0.31	0.25	0.36
$E(n)$-EPMP	0.02	0.05	**0.002**	0.24	0.17	0.32
PiNet (xyz)	0.11	0.18	0.10	0.11	**0.09**	**0.28**
PiNet (xyz+hks)	0.11	0.23	0.12	0.16	0.17	0.34
$DiffNet_{pc}$	0.12	0.18	0.09	0.16	0.15	0.34
$DiffNet_{pc}$ (hks)	**0.01**	**0.02**	0.02	0.20	0.17	0.34
$DiffNet_{pc}$ (xyz+hks)	0.10	0.16	0.07	0.12	0.13	0.33
$DiffNet_{m}$ (xyz)	0.09	0.18	0.08	**0.10**	0.11	**0.28**
$DiffNet_{m}$ (hks)	**0.01**	0.02	0.02	0.21	0.16	0.31
$DiffNet_{m}$ (xyz+hks)	0.10	0.15	0.07	0.12	0.13	0.30

(b) Acceptable reconstructions

	Antigen			Antibody		
	MCC	AUC ROC	AUC PR	MCC	AUC ROC	AUC PR
EPMP	0.02	0.02	**0.005**	0.34	0.29	0.40
$EPMP_{xyz}$	0.03	0.04	0.01	0.32	0.28	0.40
$E(n)$-EPMP	0.02	0.05	0.01	0.29	0.21	0.41
PiNet (xyz)	0.15	0.23	0.16	**0.13**	**0.05**	**0.29**
PiNet (xyz+hks)	0.16	0.27	0.17	0.19	0.13	0.34
$DiffNet_{pc}$	0.18	0.21	0.15	0.21	0.17	0.37
$DiffNet_{pc}$ (hks)	**0.003**	0.01	0.01	0.28	0.24	0.42
$DiffNet_{pc}$ (xyz+hks)	0.20	0.19	0.16	0.20	0.11	0.35
$DiffNet_{m}$ (xyz)	0.19	0.22	0.17	**0.13**	0.08	**0.29**
$DiffNet_{m}$ (hks)	0.01	**0.004**	0.01	0.29	0.22	0.41
$DiffNet_{m}$ (xyz+hks)	0.17	0.19	0.16	0.18	0.09	0.33

Alpha-Fold2 predictions

We test our model on Alpha-Fold predictions that was measured to have acceptable accuracy according to the reported comprehensive benchmark in Yin and Pierce [2024]. The complexes have a mean root-mean-square deviation (RMSD) of 13.54 Å for the antigens and of 1.92 Å for the antibody.

The Alpha-Fold results in Table 3 consistently exhibit lower values than the experimental complex results. The variance in performance is associated with the visibly distinct structure of the reconstructed antibody. However, the results in figures 5, 6, 7 and 8, show that the models provided accurate predictions for the geometry with predictions predominantly localized around the binding molecule for both paratope and epitope. The negligible drop in performance observed between reconstructions with high and acceptable accuracy aligns with the notion that the model is capturing useful protein geometry. This motivates continued exploration of techniques that utilize geometric information while also improving upon AlphaFold-like methodologies.

Additionally, in Table 4, we report the Wasserstein distance between the results on the experimental and Alpha-Fold predicted complexes.

References

James Dunbar, Konrad Krawczyk, Jinwoo Leem, Terry Baker, Angelika Fuchs, Guy Georges, Jiye Shi, and Charlotte M Deane. Sabdab: the structural antibody database. *Nucleic acids research*, 42(D1):D1140–D1146, 2014.

Jessica H Lee, Rui Yin, Gilad Ofek, and Brian G Pierce. Structural features of antibody-peptide recognition. *Frontiers in Immunology*, 13:910367, 2022.

Aaditya Ramdas, Nicolás García Trillos, and Marco Cuturi. On wasserstein two-sample testing and related families of nonparametric tests. *Entropy*, 19(2):47, 2017.

Thom Vreven, Iain H Moal, Anna Vangone, Brian G Pierce, Panagiotis L Kastritis, Mieczyslaw Torchala, Raphael Chaleil, Brian Jiménez-García, Paul A Bates, Juan Fernandez-Recio, et al. Updates to the integrated protein–protein interaction benchmarks: docking benchmark version 5 and affinity benchmark version 2. *Journal of molecular biology*, 427(19):3031–3041, 2015.

Rui Yin and Brian G Pierce. Evaluation of alphafold antibody–antigen modeling with implications for improving predictive accuracy. *Protein Science*, 33(1):e4865, 2024.

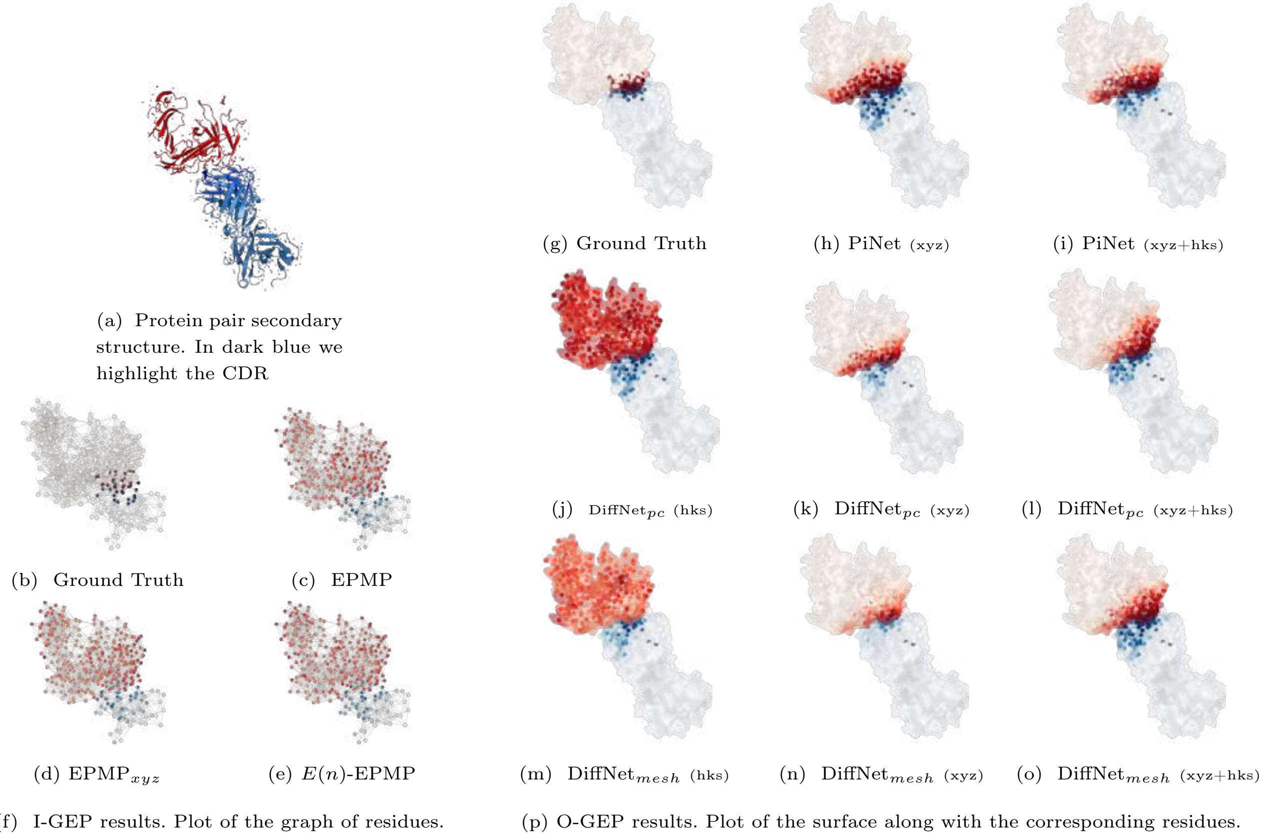

(a) Protein pair secondary structure. In dark blue we highlight the CDR

(b) Ground Truth (c) EPMP

(d) EPMP$_{xyz}$ (e) $E(n)$-EPMP

(f) I-GEP results. Plot of the graph of residues.

(g) Ground Truth (h) PiNet (xyz) (i) PiNet (xyz+hks)

(j) DiffNet$_{pc}$ (hks) (k) DiffNet$_{pc}$ (xyz) (l) DiffNet$_{pc}$ (xyz+hks)

(m) DiffNet$_{mesh}$ (hks) (n) DiffNet$_{mesh}$ (xyz) (o) DiffNet$_{mesh}$ (xyz+hks)

(p) O-GEP results. Plot of the surface along with the corresponding residues.

Fig. 3. All O-GEP and I-GEP models results for the antibody-antigen bounded complex '2fd6'. The continuous binding predictions are represented as a color gradient in red and blue for the antigen and antibody, respectively.

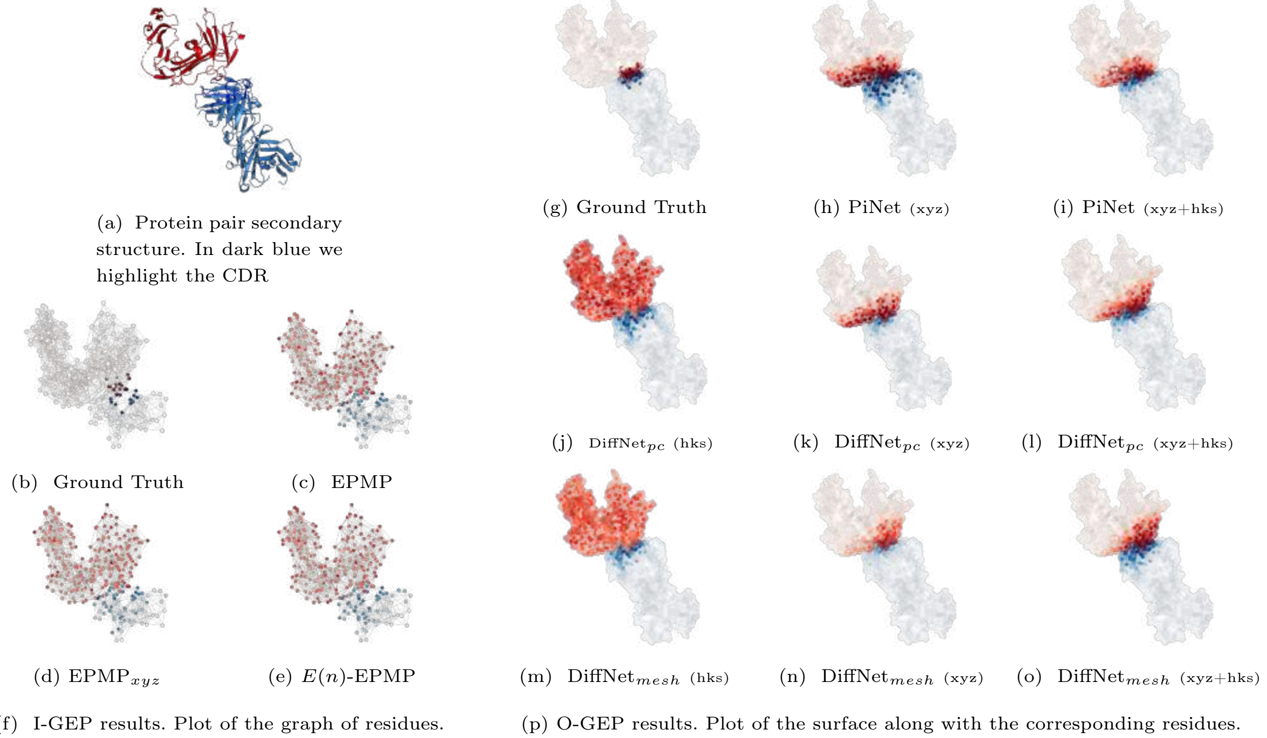

(a) Protein pair secondary structure. In dark blue we highlight the CDR

(b) Ground Truth (c) EPMP

(d) EPMP$_{xyz}$ (e) $E(n)$-EPMP

(f) I-GEP results. Plot of the graph of residues.

(g) Ground Truth (h) PiNet (xyz) (i) PiNet (xyz+hks)

(j) DiffNet$_{pc}$ (hks) (k) DiffNet$_{pc}$ (xyz) (l) DiffNet$_{pc}$ (xyz+hks)

(m) DiffNet$_{mesh}$ (hks) (n) DiffNet$_{mesh}$ (xyz) (o) DiffNet$_{mesh}$ (xyz+hks)

(p) O-GEP results. Plot of the surface along with the corresponding residues.

Fig. 4. All O-GEP and I-GEP models results for the antibody-antigen unbounded complex '2fd6'. The continuous binding predictions are represented as a color gradient in red and blue for the antigen and antibody, respectively.

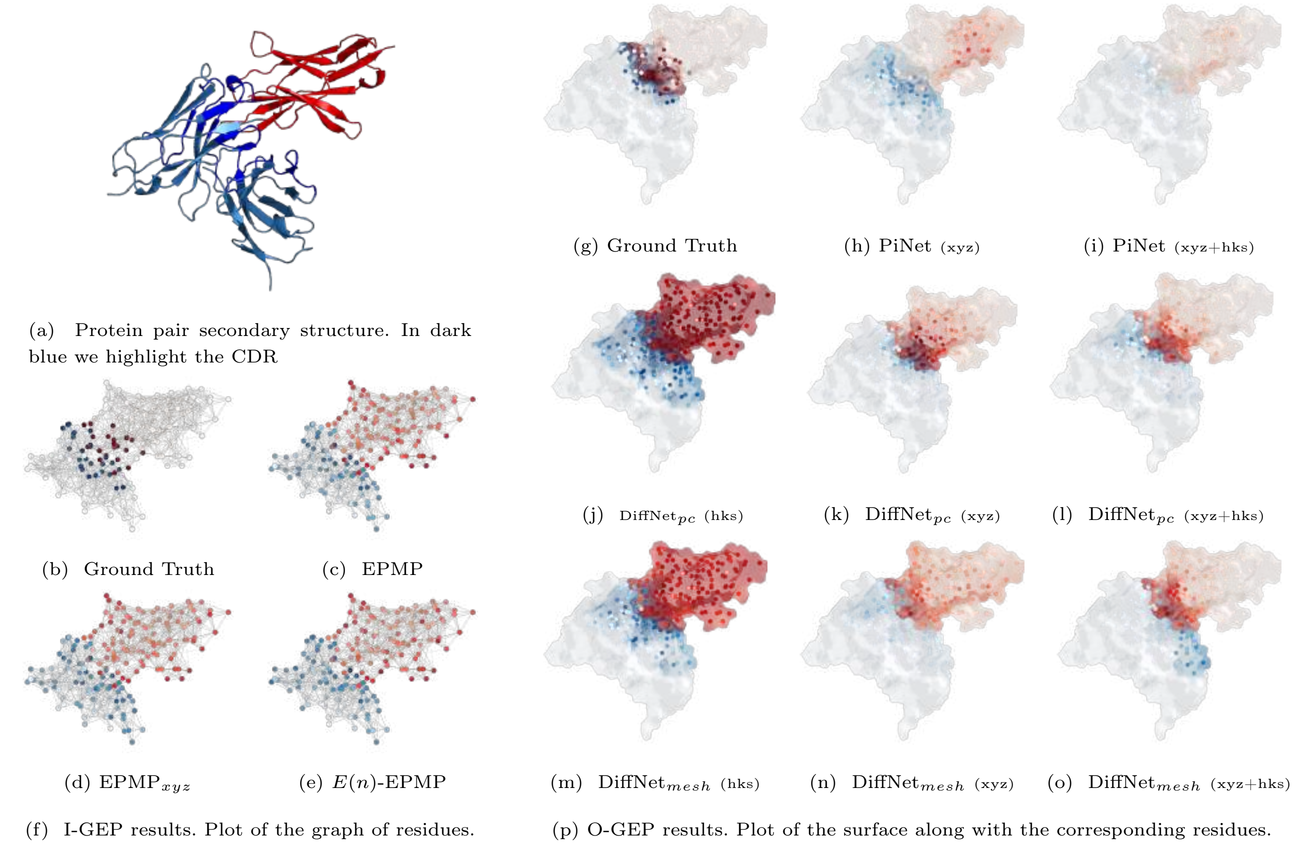

(a) Protein pair secondary structure. In dark blue we highlight the CDR

(b) Ground Truth (c) EPMP

(d) EPMP_{xyz} (e) $E(n)$-EPMP

(f) I-GEP results. Plot of the graph of residues.

(g) Ground Truth (h) PiNet (xyz) (i) PiNet (xyz+hks)

(j) DiffNet_{pc} (hks) (k) DiffNet_{pc} (xyz) (l) DiffNet_{pc} (xyz+hks)

(m) DiffNet_{mesh} (hks) (n) DiffNet_{mesh} (xyz) (o) DiffNet_{mesh} (xyz+hks)

(p) O-GEP results. Plot of the surface along with the corresponding residues.

Fig. 5. All O-GEP and I-GEP models results for the experimentally reconstructed antibody-antigen complex '7e9b'. The continuous binding predictions are represented as a color gradient in red and blue for the antigen and antibody, respectively.

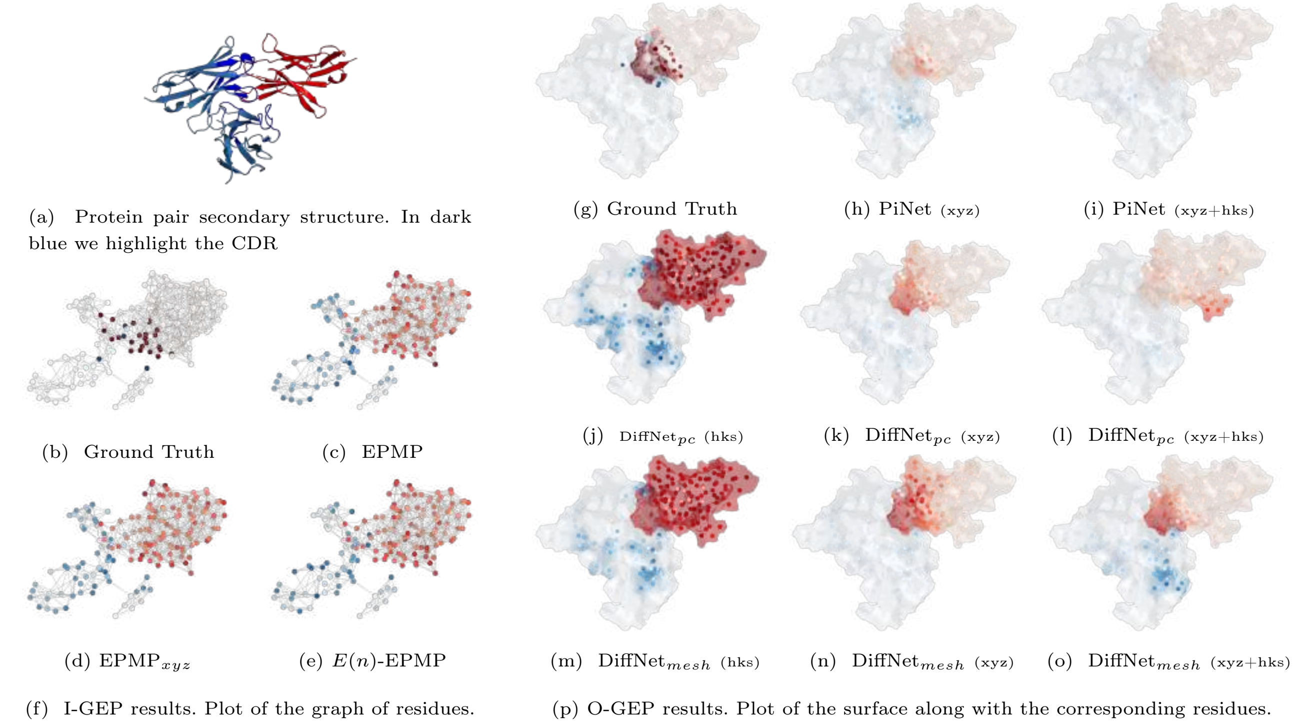

(a) Protein pair secondary structure. In dark blue we highlight the CDR

(b) Ground Truth (c) EPMP

(d) EPMP_{xyz} (e) $E(n)$-EPMP

(f) I-GEP results. Plot of the graph of residues.

(g) Ground Truth (h) PiNet (xyz) (i) PiNet (xyz+hks)

(j) DiffNet_{pc} (hks) (k) DiffNet_{pc} (xyz) (l) DiffNet_{pc} (xyz+hks)

(m) DiffNet_{mesh} (hks) (n) DiffNet_{mesh} (xyz) (o) DiffNet_{mesh} (xyz+hks)

(p) O-GEP results. Plot of the surface along with the corresponding residues.

Fig. 6. All O-GEP and I-GEP models results with high accuracy for the antibody-antigen complex '7e9b' from Alpha-Fold reconstruction. The continuous binding predictions are represented as a color gradient in red and blue for the antigen and antibody, respectively.

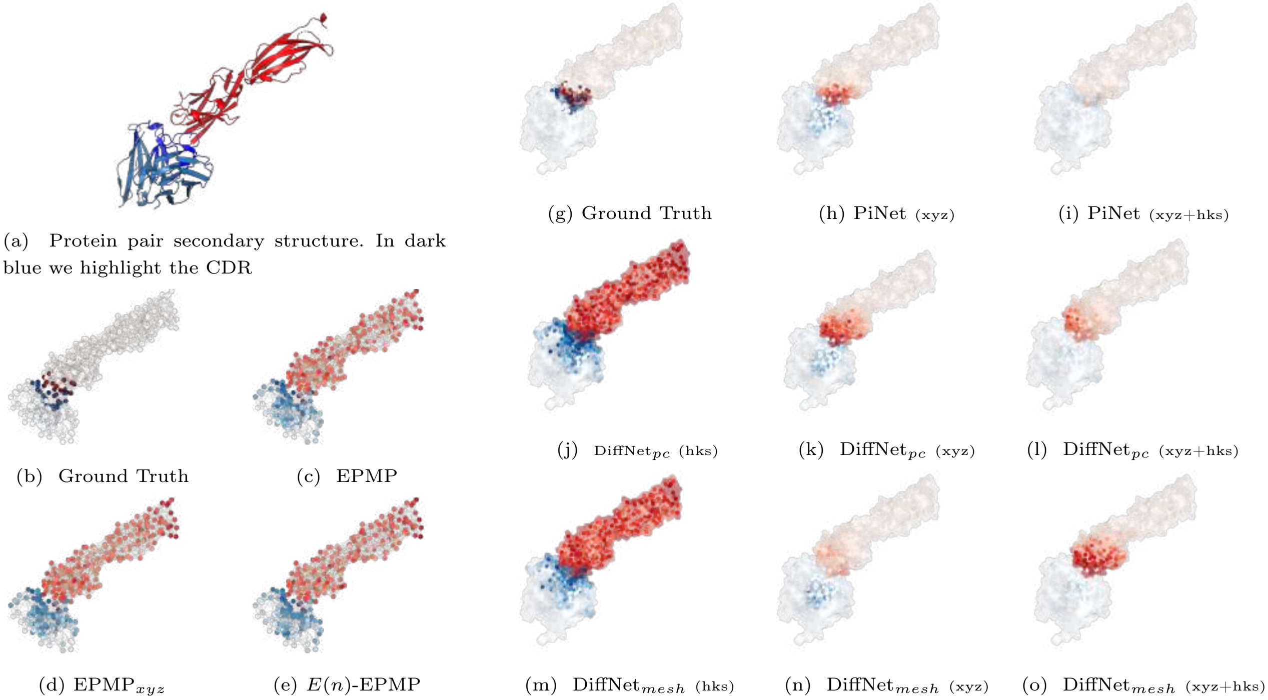

(a) Protein pair secondary structure. In dark blue we highlight the CDR

(b) Ground Truth (c) EPMP

(d) EPMP$_{xyz}$ (e) $E(n)$-EPMP

(f) I-GEP results. Plot of the graph of residues.

(g) Ground Truth (h) PiNet (xyz) (i) PiNet (xyz+hks)

(j) DiffNet$_{pc}$ (hks) (k) DiffNet$_{pc}$ (xyz) (l) DiffNet$_{pc}$ (xyz+hks)

(m) DiffNet$_{mesh}$ (hks) (n) DiffNet$_{mesh}$ (xyz) (o) DiffNet$_{mesh}$ (xyz+hks)

(p) O-GEP results. Plot of the surface along with the corresponding residues.

Fig. 7. All O-GEP and I-GEP models results for the experimentally reconstructed antibody-antigen complex '6xlq'. The continuous binding predictions are represented as a color gradient in red and blue for the antigen and antibody, respectively.

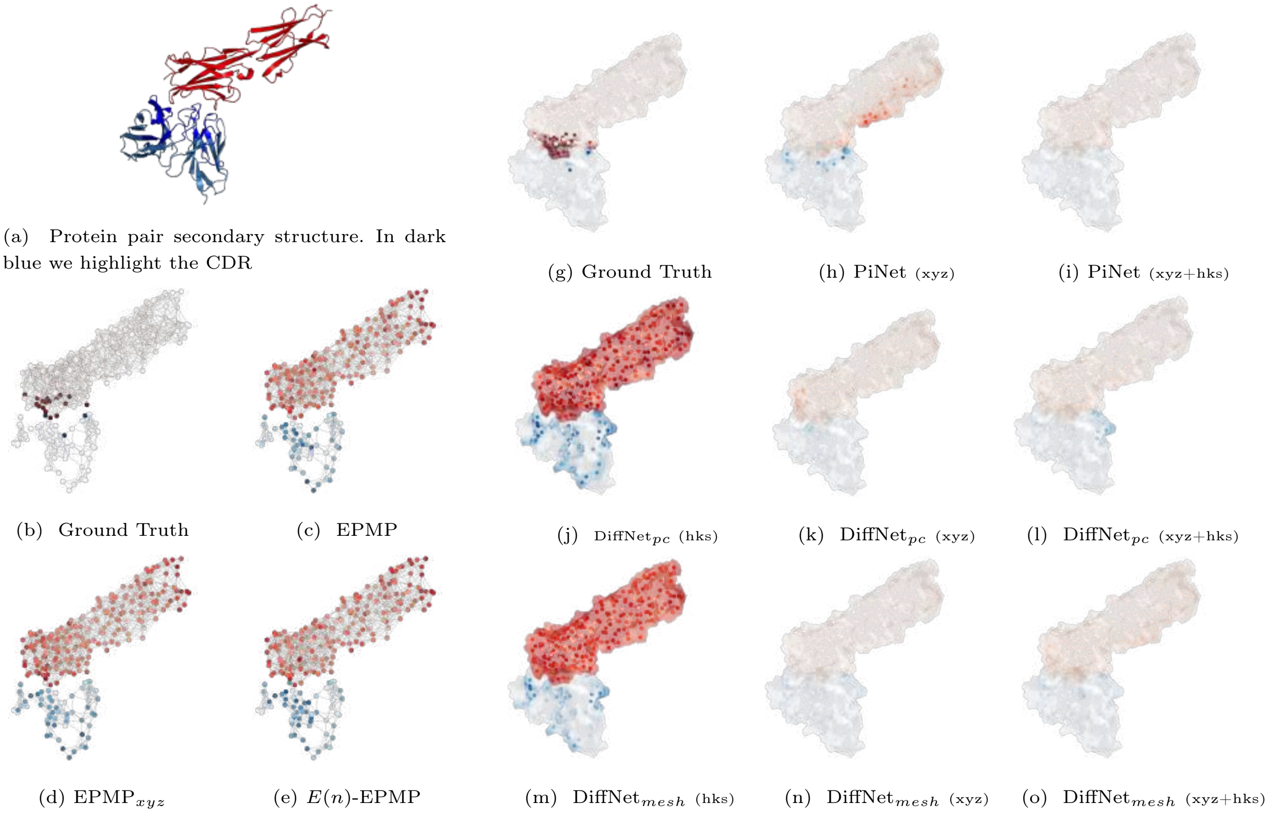

(a) Protein pair secondary structure. In dark blue we highlight the CDR

(b) Ground Truth (c) EPMP

(d) EPMP$_{xyz}$ (e) $E(n)$-EPMP

(f) I-GEP results. Plot of the graph of residues.

(g) Ground Truth (h) PiNet (xyz) (i) PiNet (xyz+hks)

(j) DiffNet$_{pc}$ (hks) (k) DiffNet$_{pc}$ (xyz) (l) DiffNet$_{pc}$ (xyz+hks)

(m) DiffNet$_{mesh}$ (hks) (n) DiffNet$_{mesh}$ (xyz) (o) DiffNet$_{mesh}$ (xyz+hks)

(p) O-GEP results. Plot of the surface along with the corresponding residues.

Fig. 8. All O-GEP and I-GEP models results with acceptable accuracy for the antibody-antigen complex '6xlq' Alpha-Fold reconstruction. The continuous binding predictions are represented as a color gradient in red and blue for the antigen and antibody, respectively.
